# Decreased expression of the Nkx2.8 gene correlates with tumor progression and a poor prognosis in HCC cancer

**DOI:** 10.1186/1475-2867-14-28

**Published:** 2014-03-30

**Authors:** Lei Qu, Biao Deng, Yue Zeng, Zhongwei Cao

**Affiliations:** 1 Department of General Surgery, Shanghai First People’s Hospital, Shanghai Jiao Tong University, 100 Haining Road, Shanghai 200080, China; 2 Department of Gastroenterology, Shanghai First People`s Hospital, Shanghai Jiao Tong University, 100 Haining Road, Shanghai 200080, China

**Keywords:** Nkx2.8, HCC, Prognosis, Clinicopathological factors, Cell proliferation

## Abstract

**Background:**

Nkx2.8 (Nk2 homeobox 8) is a novel NK-2 gene family member that has been implicated in the progression of human cancer. Its role in the progression of HCC remains unknown. In this study, we investigated the expression levels and prognostic value of Nkx2.8 in hepatocellular carcinoma.

**Methods:**

The expression of Nkx2.8 was determined by real-time quantitative RT-PCR (qRT-PCR) and immunochemistry in paired cancerous and non-cancerous tissues of 48 patients with HCC. The relationships between the Nkx2.8 expression levels, the clinicopathological characteristics and patient survival were analyzed. The effects of Nkx2.8 overexpression on cellular proliferation ability, including MTT and colony formation assays, were investigated.

**Results:**

Nkx2.8 expression was significantly downregulated in HCC cancer tissues compared with adjacent non-cancerous tissues. Further immunohistochemical analysis showed low expression of Nkx2.8 in HCC cancer tissues, and the clinicopathological analysis showed that the Nkx2.8 mRNA and protein expression levels were significantly correlated with the TNM stage (p = 0.032; p = 0.026, respectively). Kaplan–Meier survival curves revealed that lower Nkx2.8 expression was associated with a poor overall survival in HCC patients (P = 0.00172). The overexpression of Nkx2.8 in HCC cell lines inhibits cell proliferation and colony formation.

**Conclusions:**

Our data indicated that Nkx2.8 plays important roles in the development and progression of HCC and might be a valuable prognostic biomarker and potential therapeutic target for HCC.

## Introduction

Hepatocellular carcinoma (HCC) is one of the most fatal malignancies worldwide, particularly in Asia and Africa, and the incidence of HCC is increasing in Western countries because of chronic hepatitis C virus infections[1,2]. Although an increasing number of therapeutic strategies, including surgical resection, chemotherapy and liver transplantation, are now available for patients, the five-year postoperative survival rate remains poor[3,4]. Aberrant changes in genes and multiple molecular pathways might result in the malignant transformation and progression of HCC [5-8]. The key genes and molecular mechanisms involved in the disease development and progression remain unclear. It is critical to identify valuable factors for prognosis predictions and novel therapeutic strategies.

Nkx2.8 (Nk2 homeobox 8) is a novel member of the NK-2 gene family, which has been involved in tumor progression and metastasis in a variety of cancers [9-11]. Some studies have suggested that Nkx2.8 is downregulated in non-small cell lung cancer because the deletion of chromosomal DNA and Nkx2.8 overexpression inhibits cell growth and colony formation of lung cancer cells [11]. Recent reports showed that Nkx2.8 plays a potential tumor suppressor role in bladder cancer by the upregulation of p27 and inhibition of the MEK/ERK pathway activity [9]. Decreased Nkx2.8 expression is associated with the clinical stage and overall survival [9,11]. These data suggest that Nkx2.8 acts as a tumor suppressor in carcinogenesis. The expression levels and biological function of Nkx2.8 have not been studied in HCC.

In this study, we examined the mRNA and protein expression levels of Nkx2.8 in HCC tissue samples. The correlations and clinicopathological features of HCC patients were evaluated. The overall survival value of Nkx2.8 in HCC was investigated. Overexpression of Nkx2.8 significantly inhibited cell proliferation and colony formation of HCC cancer cells. These results provide new insights into the role of Nkx2.8 in the development and progression of HCC.

## Materials and methods

### Patients and tumor characteristics

Forty-eight primary HCC specimens and normal adjacent tissues were collected from patients who underwent complete resection at the Shanghai First People’s Hospital between Dec 2004 and May 2007 and were diagnosed with hepatic carcinoma (HCC) using histological diagnoses. Histological diagnoses and tumor differentiation were assayed by hematoxylin and eosin stained tumor tissue sections, according to the World Health Organization (WHO) classification guidelines (2004). Written informed consent was obtained from all of the participants involved in the study. This study was performed with the approval of the Medical Ethical Committee of Shanghai First People’s Hospital. Staging was performed according to the tumor-node-metastasis (TNM) classification of the American Joint Committee on International Union against Cancer. Tumor differentiation was defined by the Edmonson and Steiner grading system. Patient follow up occurred at our outpatient department for at least two years or until patient death. All of the tissue samples were flash-frozen in liquid nitrogen immediately after collection and stored at -80°C until use.

### Cell lines

The human HCC cell lines PLC and Hep3B were maintained in DMEM with 10% FBS (HyClone), 100 units/ml penicillin G, 100 μg/ml streptomycin sulfate at 37°C in a humidified incubator in an atmosphere of 5% CO_2_ in air.

### RNA extraction and reverse transcription polymerase chain reaction

The total RNA was isolated from HCC tissue specimens and cultured cells with the TRIzol Reagent (Invitrogen). The first-strand of cDNA synthesis was performed with 500 ng of total RNA treated with iScript™ cDNA Synthesis Kit (Bio-Rad) according to the manufacturer’s instructions. The real-time quantitative reverse-transcription polymerase chain reaction (qRT-PCR) was performed using the SYBR Green master mix (Invitrogen) according to the manufacturers’ instructions in an ABI 7900HT real-time PCR system (Life Technologies). The qRT-PCR analysis was performed in a total volume of 20 μl with the following amplification steps: an initial preheated step at 95°C for 10 min and amplification at 95°C (30 sec) and 60°C (1 min) for 40 cycles. The primer sequences were: 5′-TCCCTCACCTTCCCTCCAC-3′(sense) and 5′-CCCGCTGACAAGAATCCC-3′(antisense) for Nkx2.8; 5′-CGTCTTCCCCTCCATCGT-3′(sense) and 5′-GAAGGTGTGGTGCCAGATTT-3′(antisense) for β-actin. β-actin was used as a loading control. The Ct (threshold cycle) value of each sample was calculated from the threshold cycles with the instrument’s software (SDS 2.3), and the relative expression of Nkx2.8 mRNA was normalized to β-actin. Each assay was repeated three times.

### Immunohistochemistry

The paraffin-embedded tumor specimens were examined by two pathologists who were blinded to the clinical data. A final score was determined by reassessment on a double-headed microscope in cases of discrepancies. The tumor samples were fixed in 4% neutral formalin embedded in paraffin and cut into 4-μm-thick sections. The sections were deparaffinized in xylene and rehydrated with graded alcohol, and 3% H_2_O_2_ was applied to block the endogenous peroxide activity for 15 min at room temperature. The antigen was retrieved at 95°C in 0.01 M sodium citrate buffer (pH 6.0) for 20 min, and the sections were blocked with normal goat serum. The slides were incubated with a Nkx2.8 antibody (Prosci) in a humidity chamber at 4°C overnight. After washing, the sections were incubated with streptavidin-biotin-conjugated horseradish peroxidase and developed using 3,3′-diaminobenzidine tetrahydrochloride. Mayer’s hematoxylin was used for counterstaining. The immunostaining for Nkx2.8 was semi-quantitatively scored as ‘-’ (no signal), + (weak), ++ (moderate), +++ (strong); (++) to (+++) scores were considered positive (normal Nkx2.8 expression) and (−) to (+) scores were considered negative (abnormal Nkx2.8 expression).

### MTT assays

Transfected PLC and Huh7 cells were plated in 96-well tissue culture plates at a density of 3×10^3^/well. The MTT assay (Sigma) was used to assess the relative cell growth every 24 h. MTT (20 μl of 5 mg/ml) was added to each well, and the plates were incubated for 2 h at 37°C. DMSO (200 μl) (Sigma) was added, and the plates were shaken for 10 min to allow the formazan crystals to dissolve. The absorbance at 490 nm was measured using a Coulter Counter (Beckman Coulter). The average absorbance in each well was used to calculate the relative cell number at each time point.

### Colony formation assay

After transfection, the cells were counted and grown (500 cells/well) in six-well plates with culture medium. The culture medium was changed every three days. The number of colonies was counted only if they contained more than 50 cells at 12 days after seeding. The cells were stained with 1% crystal violet for 15 min. The ability of colony formation = (colony number)/(seeded cell number). The experiment was repeated three times, and a mean value was presented.

### Nkx2.8 cDNA plasmid and transfection

For the overexpression of Nkx2.8, we introduced the eukaryotic expression plasmid pcDNA3.1(+) containing the full-length of human Nkx2.8 cDNA. The plasmid was provided by Doctor Zhu (Fudan University, China). The empty vector was used as a negative control.

PLC and Hep3B cells were cultured in 10-cm dishes until they reached 50–60% confluence, and the transient transfections were performed using X-tremeGENE (Roche) according to the manufacturer’s protocol. Gene expression was confirmed by western blotting analysis 48 h after transfection. The cell proliferation and colony formation were assayed.

### Western blotting

The transfected cells were washed twice with ice-cold phosphate-buffered saline and lysed with RIPA buffer on ice for 30 min. After centrifugation at 12,000 rpm for 20 min at 4°C, the protein concentration of the cell lysates was measured using a BCA protein assay kit (Pierce). Forty micrograms of the protein samples were resolved on a 10% SDS denatured polyacrylamide gel and transferred onto a 0.45-μm PVDF membrane (Millipore, Bedford, MA, USA). The membranes were blocked with 5% skim milk in TBS + 0.05% Tween™ 20 for 1 h at room temperature and incubated with an Anti-Nkx2.8 (Abcam) antibody or Anti-β-actin (Santa Cruz) antibody overnight at 4°C. The target proteins were probed with an IgG peroxidase-conjugated secondary antibody (KangChen Bio-tec, China) followed by an enhanced chemiluminescence reaction (Pierce) and quantified by the DNR Bio-Imaging System. β-actin was used as the endogenous control to normalize the relative expression level of Nkx2.8. All of the experiments were repeated three times, and a mean value was presented.

### Statistical analysis

All of the data were shown as the mean ± SD for n ≥ three experiments. Paired adjacent HCC tissues and HCC cancer samples were compared using Student’s t test. The Chi-square (χ2) analysis was used to examine the possible correlations between Nkx2.8 expression profiles and clinicopathological factors. The probability of patient survival was estimated using the Kaplan–Meier method. P < 0.05 was considered statistically significant.

## Results

### Nkx2.8 mRNA expression was analyzed with qRT-PCR

**Figure 1:**
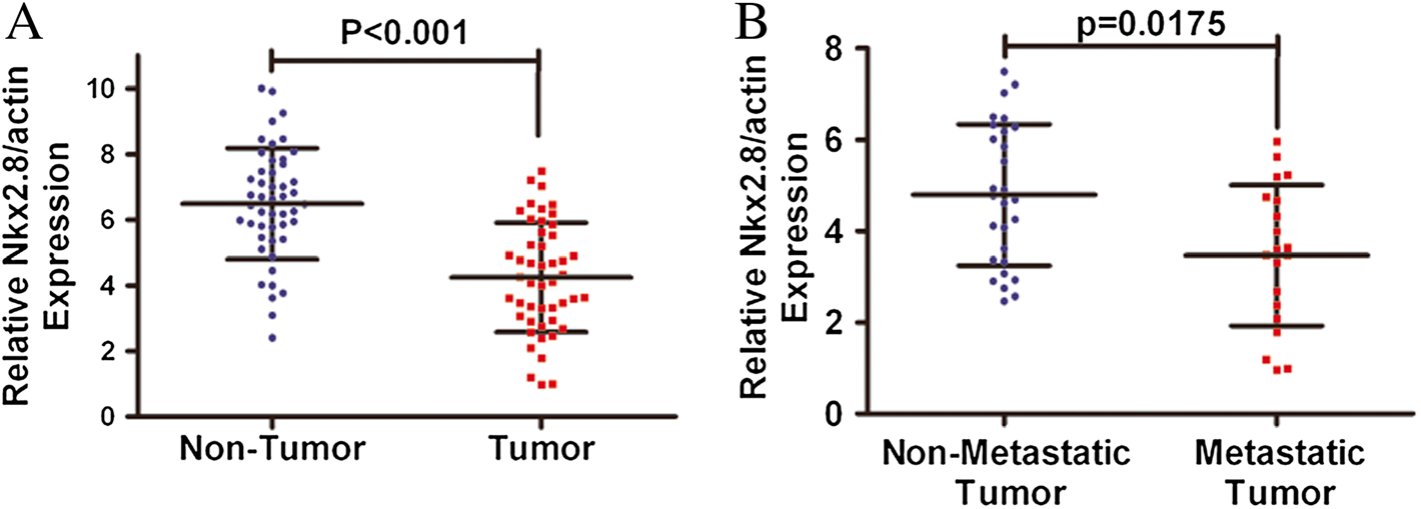
**The mRNA expression levels of Nkx2.8 in HCC cancer tissues. (A)** The Nkx2.8 mRNA expression level was lower in the tumor tissues compared with the adjacent non-malignant tissues as examined by real-time RT-PCR analysis (mean ± SD; n = 48; P < 0.001). **(B)**. The Nkx2.8 expression in non-metastasis (n = 28) and metastasis (n = 20) patients was quantified by qPCR and normalized to β-actin (mean ± SD; P < 0.0175).

The mRNA levels of Nkx2.8 in human HCC were determined using qRT-PCR in 48 pairs of resected specimens (tumor tissue samples and corresponding adjacent normal tissue samples). As shown in Figure 1A, the Nkx2.8 mRNA levels were significantly reduced in 87.3% (41 out of 48 patients) of HCC tumor tissues compared with the matched adjacent non-tumor tissue samples (P < 0.001). Within the tumor tissues, the transcriptional levels of Nkx2.8 in the patients without metastasis were obviously downregulated compared with the levels in the patients with metastasis (P < 0.0175) (Figure 1B). These results suggest that Nkx2.8 is decreased in HCC tissues, which indicates that Nkx2.8 might be involved in HCC carcinogenesis.

### Immunohistochemical analysis of NKX2.8 expression in HCC tissue samples

**Figure 2:**
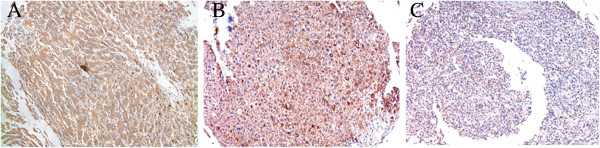
**Immunohistochemical staining of Nkx2.8 in primary HCC. (A)** A representative image of Nkx2.8 staining in adjacent non-cancerous tissues is shown (100× microscopic field). **(B)** A representative image of Nkx2.8 staining in colorectal cancer tissues is shown (100 × microscopic field). **(C)** Nkx2.8 is negative in the cancer cells of HCC with metastasis (100 × microscopic field).

To confirm the expression and location of Nkx2.8, we analyzed the Nkx2.8 protein subcellular localization in the 48 above-mentioned surgical tumor samples using immunohistochemical methods. We assessed the Nkx2.8 expression based on the staining intensity and the percentage of tumor cells in a semiquantitative manner. The positive expression of Nkx2.8 was mainly localized to the cytoplasm. Of the 48 HCC samples, 20 (41.7%) showed high Nkx2.8 expression (Nkx2.8++ or Nkx2.8+++), and the remaining 28 (58.3%) displayed low Nkx2.8 expression (Nkx2.8- or Nkx2.8+) (Figure 2, Table 1). Adjacent normal tissue samples showed the strongest accumulation of Nkx2.8 protein.

**Table 1:** Associations between NKX2-8 expression levels and clinicopathologic parameters of HCC

**Variables**	**NKX2-8 mRNA expression**		***P***	**NKX2-8 protein expression**		***P***
	**Low**	**High**		**Negative**	**Positive**	
	**(n = 20)**	**(n = 28)**		**(n = 26)**	**(n = 22)**	
**Gender**						
Female	3	4	0.572	3	4	0.615
Male	17	24		23	18	
**Age**						
≤50	11	13	0.734	14	9	0.643
>50	9	15		9	13	
**Preoperative AFP (ng/mL)**						
≤20	7	12	0.126	11	8	0.263
>20	13	16		15	14	
**Liver cirrhosis**						
No	4	8	0.101	3	9	0.092
Yes	16	20		23	13	
**Tumor diameter (cm)**						
≤5	14	21	0.164	17	18	0.076
>5	6	7		9	4	
**Tumor encapsulation**						
None	11	13	0.375	11	13	0.469
Complete	9	15		9	9	
**Clinical TNM stage**						
I-II	14	23	0.032*	17	20	0.026*
III-IV	6	5		9	2	

One-way Analysis of Variance (ANOVA) test was performed.

*Indicate a significant difference (P < 0.05) by Student’s t-test.

### Association between NKX2-8 and clinicopathological factors and its relationship with the clinicopathological features

To evaluate the relationship between the mRNA or protein levels of Nkx2.8 and HCC progression, we analyzed the correlation between low expression levels of Nkx2.8 and the clinicopathological parameters of liver cancers. The data are summarized in Table 1. We found a significant correlation between Nkx2.8 expression and the TNM stage at the mRNA (p = 0.032) and protein levels (p = 0.026). Among the 48 HCC patients, the expression level of Nkx2.8 showed no difference among age, gender, liver cirrhosis, tumor size, tumor encapsulation or AFP levels at the mRNA or protein levels (p > 0.05).

### Expression of Nkx2.8 based on qRT-PCR in relation to prognosis

**Figure 3:**
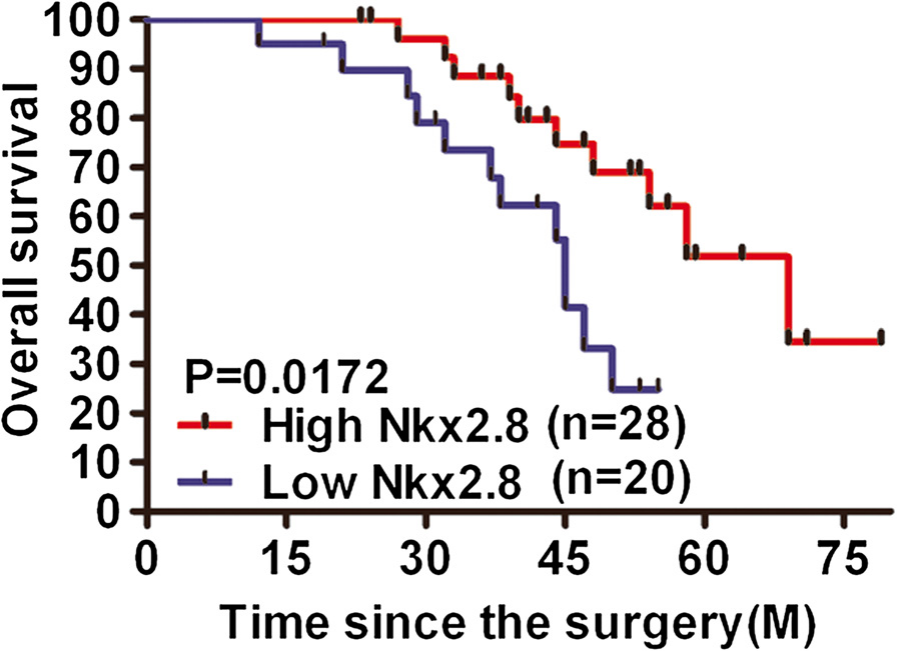
**Nkx2.8 expression correlates with patient survival.** Kaplan–Meier curves show the overall survival of HCC patients after surgical resection based on the median value of the Nkx2.8 expression level.

We investigated whether the expression of Nkx2.8 was associated with the prognosis of 48 HCC whose progress was monitored after surgery resections. The patients were categorized into two groups in terms of the median Nkx2.8 expression level. The Kaplan-Meier survival analysis and log-rank test showed that the overall survival rate was significantly improved in the Nkx2.8 high expression group (median value = 69 months; n = 28) compared with the low expression group (median value = 45 months; n = 20) (Figure 3). Our data showed that an aberrant Nkx2.8 expression profile was correlated with tumor progression and a poor prognosis in HCC.

### Nkx2.8 inhibits tumor cell proliferation in vitro

**Figure 4:**
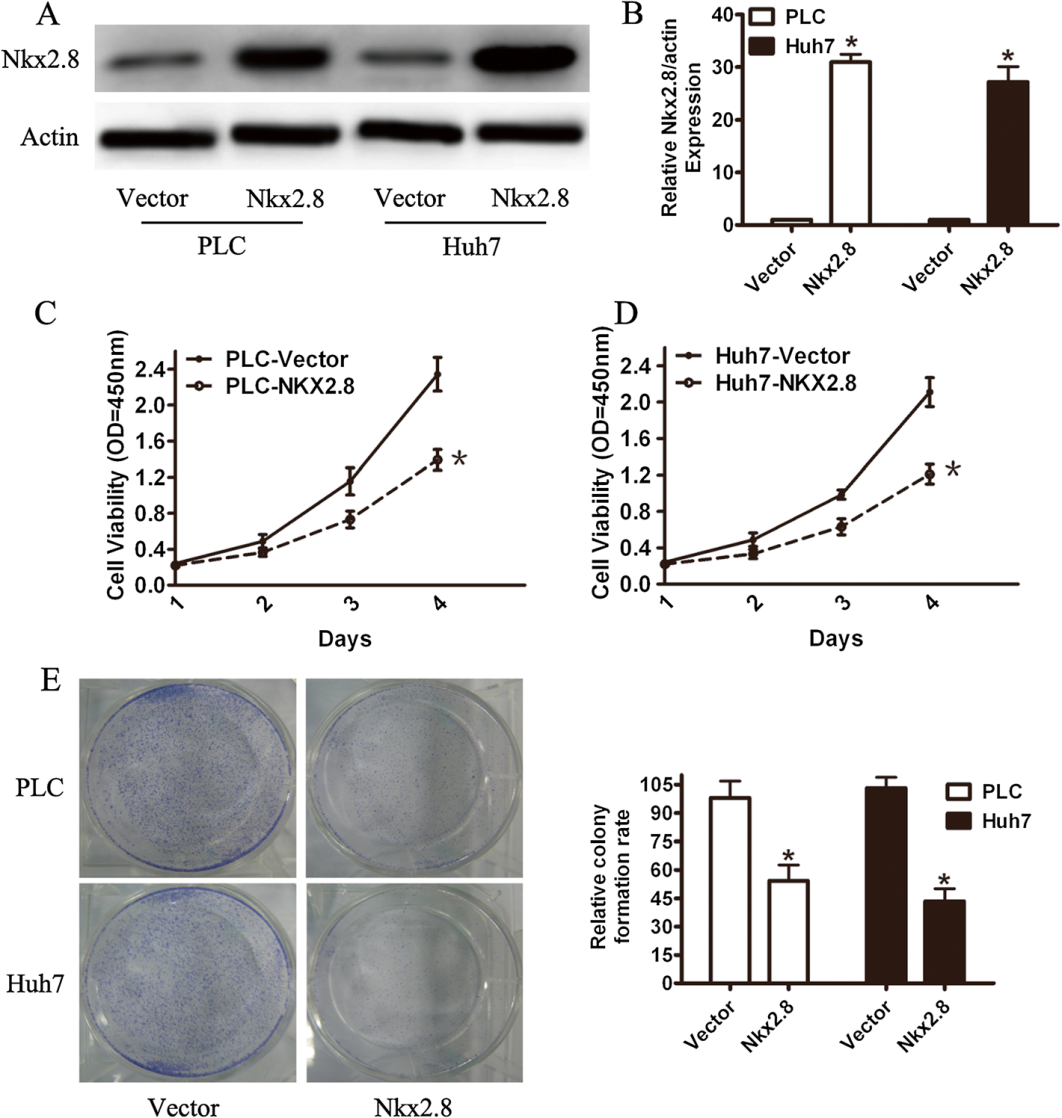
**Effect of Nkx2.8 overexpression on HCC cell function. (A, B)** The detection of Nkx2.8 overexpression in PLC and Huh7 cells by WB and q-RT-PCR 48 h after transfection, respectively. **(C, D)** The cell proliferative activities were determined in PLC and Huh7 cells by a MTT assay. Each column represents the mean ± S.D. for three independent experiments. **(E)** A representative result of the colony formation assays for the effects of Nkx2.8 on the in vitro proliferation abilities of PLC and Huh7 cells. The results represent the mean ± S.D. for four independent experiments. *p < 0.05.

The above results showed that low Nkx2.8 expression was associated with a poor prognosis in HCC patients, but the underlying biological mechanisms remain unclear. We investigated the function of Nkx2.8 by exogenously overexpressing Nkx2.8 in PLC and Huh7 cells. Nkx2.8 overexpression was verified by western blotting (Figure 4A) and qRT-PCR (Figure 4B) at 48 h after transfection. The empty vector was used as a negative control. Cellular proliferation was assessed using a MTT assay and clonogenic assays. We observed overexpression of Nkx2.8, which was associated with a lower proliferation rate in the PLC (Figure 4C) and Huh7 (Figure 4D) cancer cell lines, especially at time points later than 48 h. To further confirm the role of Nkx2.8 in the growth of HCC cancer, we performed colony formation assays. The colony formation assays showed that the overexpression of Nkx2.8 significantly decreased the number and size of colonies formed compared with the vector cells after two weeks (Figure 4E). These results showed that the overexpression of Nkx2.8 significantly inhibited HCC cancer cell proliferation.

## Discussion

HCC is one of the most deadly human malignancies diagnosed worldwide [12]. Many risk factors, including hepatitis B or C viral infection, alcohol consumption, aatoxinB1 and genetic alterations, have been identified as causes of HCC pathogenesis [5,13-15]. It is well known that genetic dysfunction leading to the activation of oncogenes and/or the inactivation of tumor suppressors is involved in transformation and tumor progression [16,17]. A number of molecules, such as miR-29a [18,19], DKK1 [20] and osteopontin [21], have been shown to have effective clinical significance for predicting HCC prognosis, but the prognosis for HCC remains poor. Searching for valuable prognostic biomarkers for HCC has been attracting more interest. In the current study, for the first time, we found that low Nkx2.8 expression levels were related to a poor prognosis and advanced stages of HCC, suggesting that Nkx2.8 might be involved in the progression of HCC.

Nkx2.8, located in the 14q13.3 region, has been reported to act as a tumor suppressor in bladder cancer, esophageal cancer, lung cancer and some other malignant tumors [9-11]. Forced expression of Nkx2.8 was found to inhibit the growth of lung cancer cells [11]. Prior studies showed that n Nkx2.8 in HCC cell lines led to inhibition of proliferation in vitro. The discrepancy may be the different tissues. A recent study showed that low expression levels of Nkx2.8 in ESCCs inversely correlate with progression, and Nkx2.8 suppressed NF-kB activation by directly targeted the AKIP1 promoter; silencing Nkx2.8 promotes ESCC cell proliferation and angiogenesis in vitro and in vivo [10]. Consistent with the previous study, we found that Nkx2.8 expression was significantly decreased at the mRNA and protein levels in the tumor tissue samples compared with the expression levels in the paired adjacent non-tumor tissue samples. The expression of Nkx2.8 in patients with metastases was significantly lower than in patients without metastases [22]. Some studies have found that coactivation of TTF-1 and Nkx2.8 was associated with a poor prognosis and resistance to cisplatin [23]. These study results indicate that Nkx2.8 might serve as a tumor suppressor in HCC.

To further confirm the expression of Nkx2.8, we used IHC to determine its expression in the same patients. Consistent with the results of the mRNA, a significant decline in the expression of Nkx2.8 was observed in the HCC tissue samples compared with the adjacent non-tumorous tissues and especially those patients with highly metastatic potential. Low expression levels of Nkx2.8 were significantly correlated with the advanced clinical stage at the mRNA and protein levels. Low expression levels of Nkx2.8 were closely connected with poor overall survival by the Kaplan-Meier survival analysis. A similar result has been reported for bladder cancer [9]. These data suggest that Nkx2.8 is involved in the progression of HCC, and Nkx2.8 might be used as a marker to identify subsets of HCC patients.

To further study the effects of Nkx2.8 on cell growth, exogenous expression of Nkx2.8 inhibited the proliferation of HCC cells in a MTT assay and a colony formation assay. Our results supported the downregulation of Nkx2.8 in HCC tissue samples by qRT-PCR and immunohistochemistry, but more tissue samples are needed to confirm these results. The antibody reactivity for the retrieved antigens for IHC had interference. The discrepancy between the mRNA and protein expression levels of Nkx2.8 in the same tissue samples might be a result of post-transcriptional regulation.

Our data provide the first evidence that the mRNA and protein levels of Nkx2.8 are downregulated in HCC tissues. Our study provides compelling clinical evidence that Nkx2.8 could be used as an independent prognostic marker for the overall survival and is strongly correlated with TNM stage in HCC. The functional role of Nkx2.8 in cancer cell proliferation indicates that Nkx2.8 might be used as a new biomarker and a potential therapeutic target for HCC. Further investigation on the mechanism by which Nkx2.8 is involved in the development and progression of HCC are required.

## Competing interests

The authors declare that they have no competing interests.

## Authors’ contributions

QL carried out the experiment of cell lines. DB carried out the clinical data of the paper. ZY and ZC both carried out the design of the experiment. All authors read and approved the final manuscript.
